# Thomas E. Starzl, M.D., Ph.D—the *Sui Generis* Medical Pioneer and Mentor

**DOI:** 10.3389/frtra.2025.1688201

**Published:** 2025-11-26

**Authors:** John J. Fung, Merit Remzi

**Affiliations:** 1 Department of Surgery, The University of Chicago, Chicago, IL, United States; 2 Journalism Department, Horace Mann Institute, New York, NY, United States

**Keywords:** Thomas Starzl, organ transplantation, cyclosporine, tacrolimus, history

## Abstract

As the “Father of Modern Transplantation”, Dr. Starzl pioneered every aspect of organ transplantation: immunosuppression, organ procurement and preservation, tissue matching, surgical transplant technology, and the operational management of the transplant team. His work paved the way for heart, lung, pancreas, intestinal, liver, and kidney transplantation and opened doors to understanding immune regulation of a number of acquired and inherited disorders. Dr. Starzl’s contributions to the scientific literature, in a span of 60 years, are nothing short of remarkable—2,872 publications placing him at the top of scientific citations according to the Institute of Scientific Information. Dr. Starzl was a man of unique vision, enthusiasm, and persistence; many of his ideas were considered revolutionary and radical—stimulating opposition and criticism. He called upon an inner strength, likely entrenched from his small-town upbringing, to persist in spite of adversity and promote social and medical acceptance of transplantation. Through his tireless efforts he educated scientists, other professionals, and the public. He was involved in all of the controversies of organ donation, from the use of non-heart beating donors, to living donors, to brain dead donor and to xenotransplantation (animal-to-human transplantation).

“With determination and irresistible resolve, Thomas Starzl advanced medicine through his intuition and uncanny insight into both the technical and human aspects of even the most challenging problems. Even more extraordinary was his ability to gift that capacity to those around him, allowing his students and colleagues to discover the right stuff within themselves. Nobody who spent time with Thomas Starzl could remain unaffected.”

Joy, Ravi and Timothy Starzl—Eulogy—March 7, 2017

As an undergraduate student at Johns Hopkins University, I read with great interest a chapter on liver transplantation written by Thomas Starzl in 1975 in the book “Immunobiology” edited by Robert Good, PhD. In the chapter, he described the rejection of a recently transplanted liver in a pediatric recipient, requiring retransplantation. Nine years later, I had the opportunity of meeting Dr. Starzl in person, as the Visiting Professor at the University of Rochester. My background in immunology and studies of OKT3, then an experimental agent, led to an invitation to join Dr. Starzl at the University of Pittsburgh as a clinical research fellow in 1984. During that two-year fellowship, I worked closely with Dr. Starzl as well as the human immunology group at the University of Pittsburgh, Drs. Adrianna Zeevi and Rene Duquesnoy, as well as Dr. Anthony “Jake” Demetris, then a pathology resident, forming life-long ties and friendships. These experiences would prove to be the defining period in my career and set me on the path to becoming a transplant surgeon. I was blessed to have worked with Dr. Starzl for 20 years, remaining a close colleague and confidant spanning a 35-year period (Figure 1).

**Figure 1:**
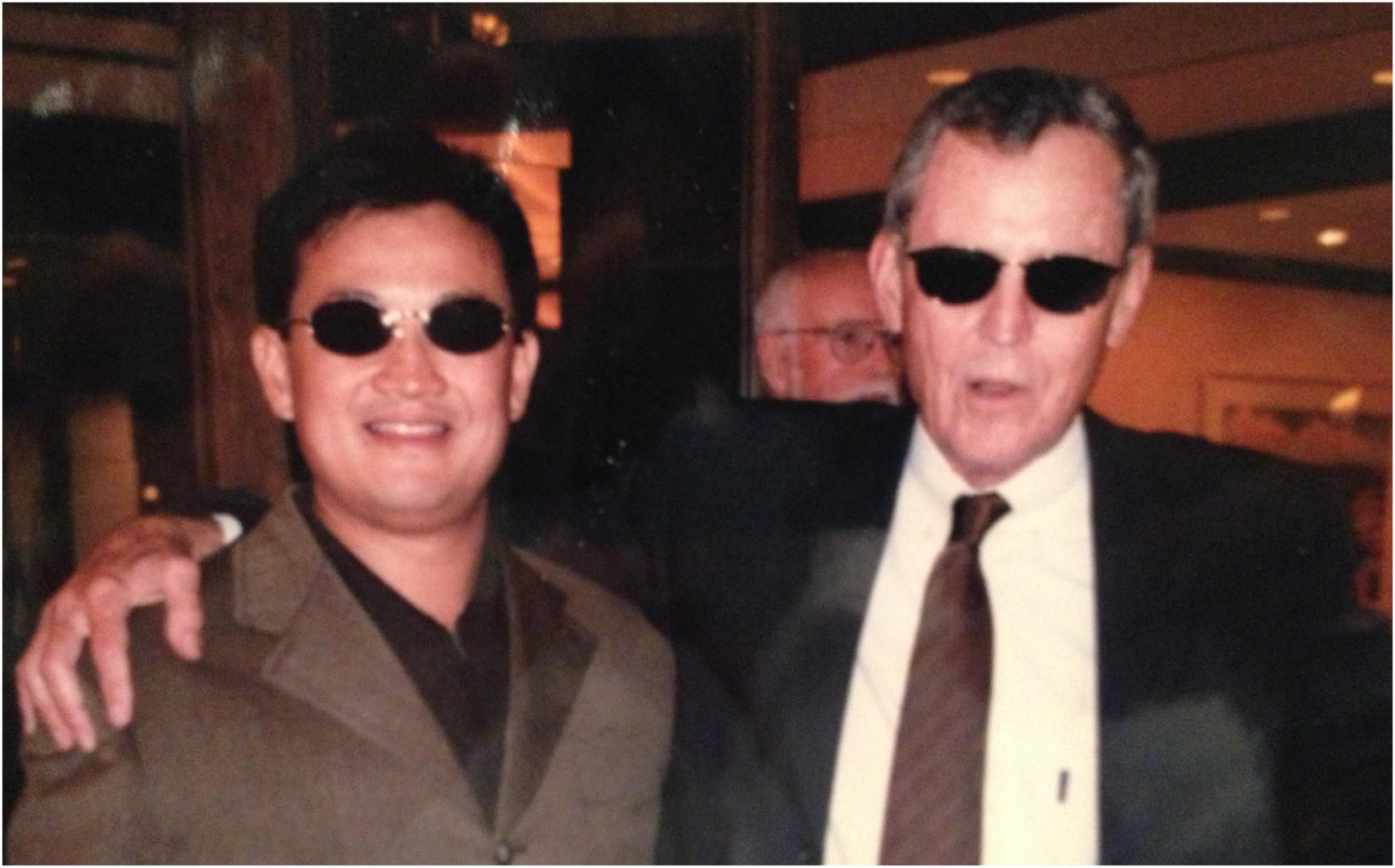
Thomas Starzl with John Fung at fundraising event, 1997 in Pittsburgh, PA.

Born in LeMars, Iowa, in 1927, Thomas E. Starzl went on to receive his undergraduate degree in biology at Westminster College in 1947. He then received his MD-PhD dual doctoral degree in neurophysiology at Northwestern University in 1952. His education formed a crucial foundation for his later scientific accomplishments, which include his mapping of the reticular activity system, an achievement that remains highly cited. Between 1952 and 1959, he trained in general and thoracic surgery at Johns Hopkins University, the University of Miami, and Northwestern University. In 1959, he was named a Markle Scholar in Medical Science, a distinction given to a small group of promising young physicians in academic medicine. Subsequently, he joined the Northwestern medical faculty (1958–61) before moving to the University of Colorado School of Medicine as Associate Professor of Surgery in December 1961. He went on to serve as the Chief of Surgery at the Denver Veterans Administration Hospital (1961–72) and Chairman of the University of Colorado Department of Surgery (1972–80). Less than three years after his arrival at Colorado, he was promoted to Professor of Surgery.

Although many experts believed that successful kidney transplantation was only possible between monozygotic twins, Dr. Starzl demonstrated otherwise by successfully transplanting kidneys between non-monozygotic twins, preventing immunologic rejection by combining azathioprine (Imuran) with corticosteroids. In 1963, he published his findings on the first series of successful allogeneic kidney transplants, in which he used organs from a broad range of donors. Applying the lessons from these groundbreaking kidney transplants and research on liver physiology, Dr. Starzl successfully performed the first human liver transplant in 1963 at the University of Colorado, adding yet a second milestone to an already monumental medical career. Over the course of his tenure, Dr. Starzl and his team performed 1,000 + kidney and 100 + liver transplants at Colorado General and Denver Veterans Administration hospitals.

Beyond his herculean achievements in the operating room, Dr. Starzl pioneered many immunosuppressive strategies; he implemented anti-lymphocyte globulin (ALG) and, later, cyclosporine in conjunction with steroids, innovations that shifted transplantation from an experimental pursuit to a viable therapy for patients with end-stage liver, kidney, and heart disease. Dr. Starzl's research ultimately laid the foundation for his move to the University of Pittsburgh in 1981 (Figure 2).

**Figure 2:**
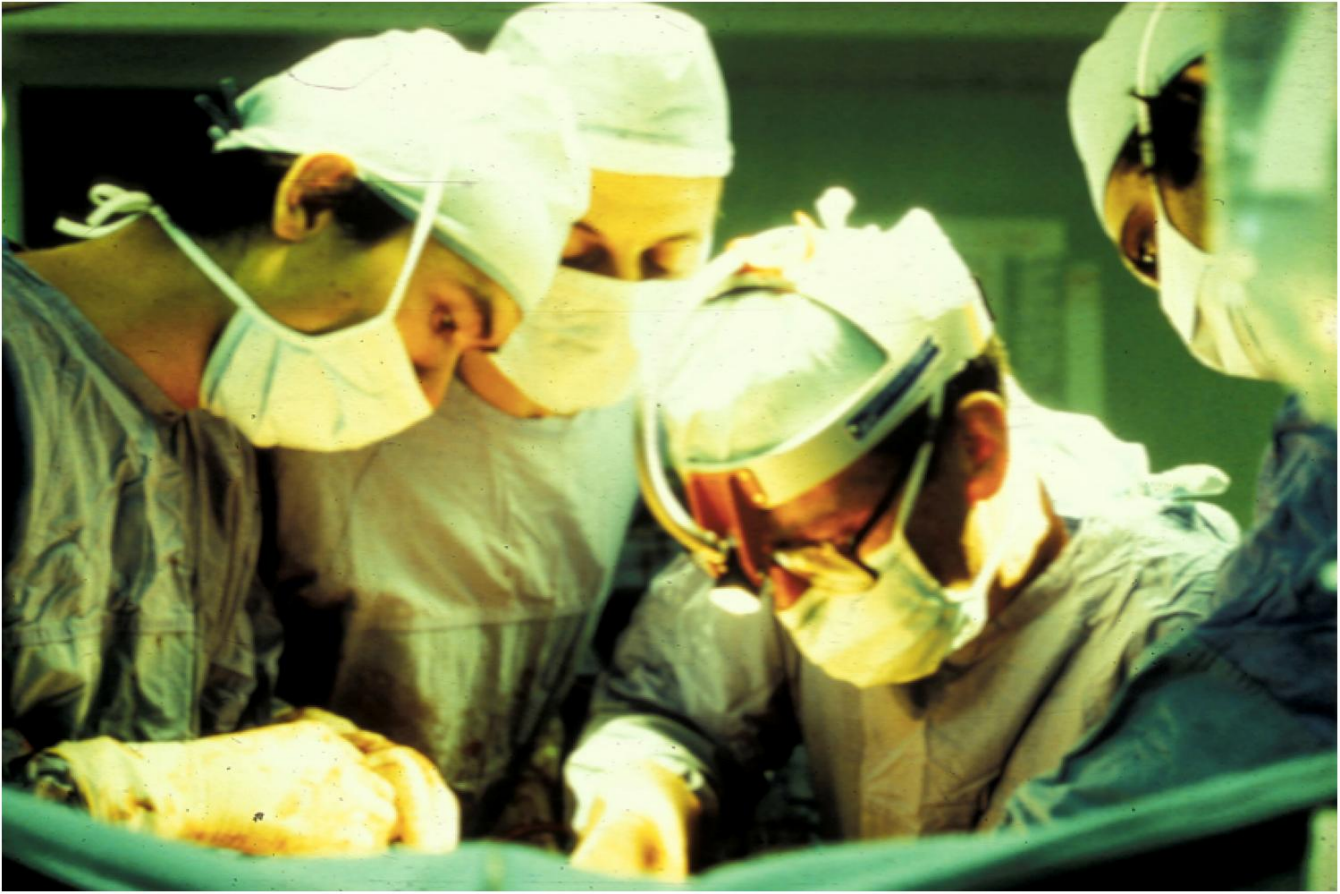
Thomas Starzl performing liver transplant at the University of Pittsburgh in 1983.

Dr. Starzl was recruited by Henry Bahnson, MD, the Chairman of Surgery the University of Pittsburgh School of Medicine. Until 1991, Dr. Starzl served as Chief of Transplantation Services at Presbyterian University Hospital (now UPMC Presbyterian), Children's Hospital of Pittsburgh, and the Veterans Administration Hospital in Pittsburgh, overseeing what became the largest and busiest transplant program in the world. He then assumed the title of Director of the University of Pittsburgh Transplantation Institute, a post that permitted his full attention to research.

In 1989, following three years of pre-clinical work, Dr. Starzl introduced the first human use of FK506 (tacrolimus), a powerful new anti-rejection drug. This breakthrough revolutionized transplant medicine, dramatically improving graft and patient survival, and making successful intestinal transplantation possible for the first time. Tacrolimus was approved by the U.S. Food and Drug Administration in 1994 and has since become the dominant immunosuppressant worldwide.

The institute, later renamed in his honor, also advanced xenotransplantation by performing baboon-to-human liver transplants and developing the first double alpha-galactosyl transferase knock-out pigs, which underpin current clinical efforts in the field of xenotransplantation. Still, Dr. Starzl's primary scientific focus was on immune tolerance and chimerism—the coexistence of donor and recipient cells as the mechanism for organ acceptance.

Dr. Starzl received more than 220 honors during his career, among them the David M. Hume Memorial Award (1978), the American Surgical Association's Medallion for Scientific Achievement (1990), the William Beaumont Prize (1991), the Peter Medawar Prize (1992), the King Faisal International Prize for Medicine (2001), the National Medal of Science (2004), the Lasker Award (2012), and the Benjamin Franklin Medal of the American Philosophical Society (2016) (1).

He was a member of more than 60 professional organizations, including serving as President of the Transplantation Society, founding president of both the American Society of Transplant Surgeons and the Transplant Recipients International Organization (TRIO). In 1992, he became one of only five American physicians elected to the French National Academy of Medicine. Over his career, he gave more than 1,400 presentations at international meetings, served on 40 editorial boards, and published over 2,300 scientific papers, along with four books and 300 book chapters.

In 1999, the Institute for Scientific Information recognized him as the world's most cited clinical scientist. Additionally, the book, 1,000 People: Ranking of Men and Women who Shaped the Millennium (A.H. Gottlieb et al, Kodansha Co., Japan), placed Dr. Starzl 213th on its list of those whose contributions have significantly most influenced history's progress between 1,000 A.D and 2,000 A.D. Furthermore, his memoir, The Puzzle People: Memoirs of a Transplant Surgeon (1992), was named by the Wall Street Journal as one of the five best doctor's memoirs of the 20th century.

When not working or traveling, Dr. Starzl had a penchant for sports. Having played baseball, basketball and football in high school, he came to Pittsburgh during a period where the Pittsburgh sports team were doing exceptionally well. He particularly liked going to the Pirates baseball games, where he could only be reached by posting an announcement on the scoreboard. In the late 1990's, through a variety of coincidences, the Pittsburgh Steelers became closely aligned with the Starzl Transplantation Institute, assisting with fundraising, organ donor card drives and hosting a number of celebratory events, such as Dr. Starzl's 90th birthday event (see Figure 3).

**Figure 3:**
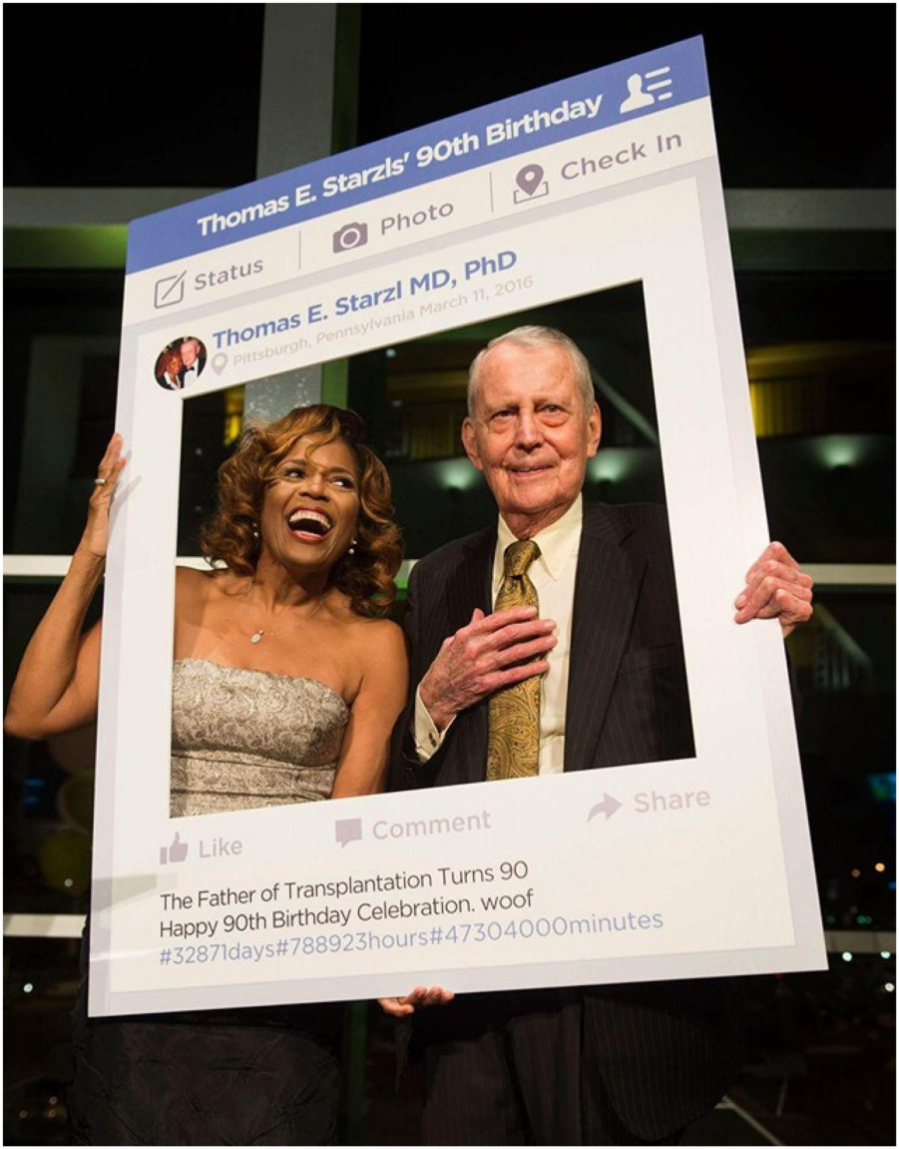
Thomas Starzl celebrating his 90th birthday at the Heinz Arena in Pittsburgh, PA along with his wife, Joy.

At a personal level, Dr. Starzl had three children from his first marriage to Barbara—Timothy, Rebecca and Thomas. He had one grandson, Ravi, who received his PhD at Carnegie Mellon University. Prior to his move to Pittsburgh, he married Joy Conger and both lived in their home near the University campus for more than 30 years with their dogs. The dogs could often be found accompanying him at work—often referred to as the Pizza Hut office (as it was located above the Pizza Hut in Oakland), where his long-time dedicated office staff, Terry Mangan and Katie Benedetti could be found (Figure 4).

**Figure 4:**
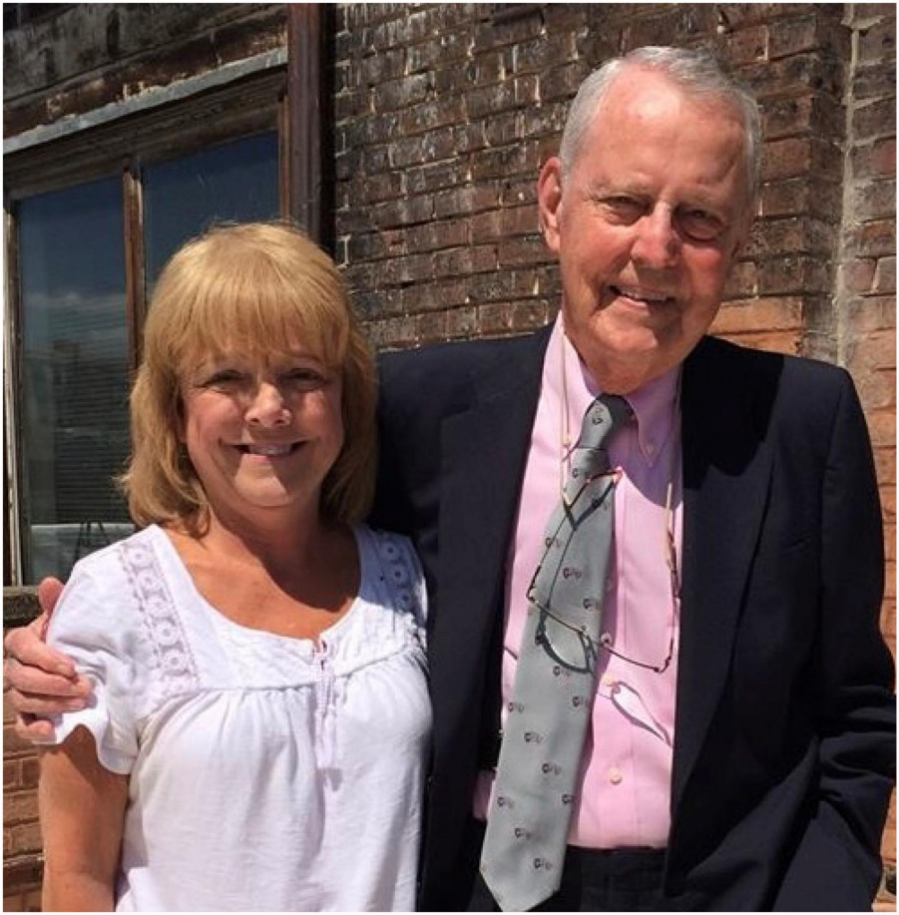
Terry Mangan was Dr. Starzl's personal assistant for more than 35 years.

Dr. Starzl died at home at the age of 90, on March 4, 2017 after a brief illness—his memorial service was conducted at Heinz Chapel at the University of Pittsburgh, where over 500 guests paid their respects in person. In honor of his service to the University of Pittsburgh, a memorial figure of him was dedicated outside of Heinz Chapel on the university campus (Figure 5).

**Figure 5:**
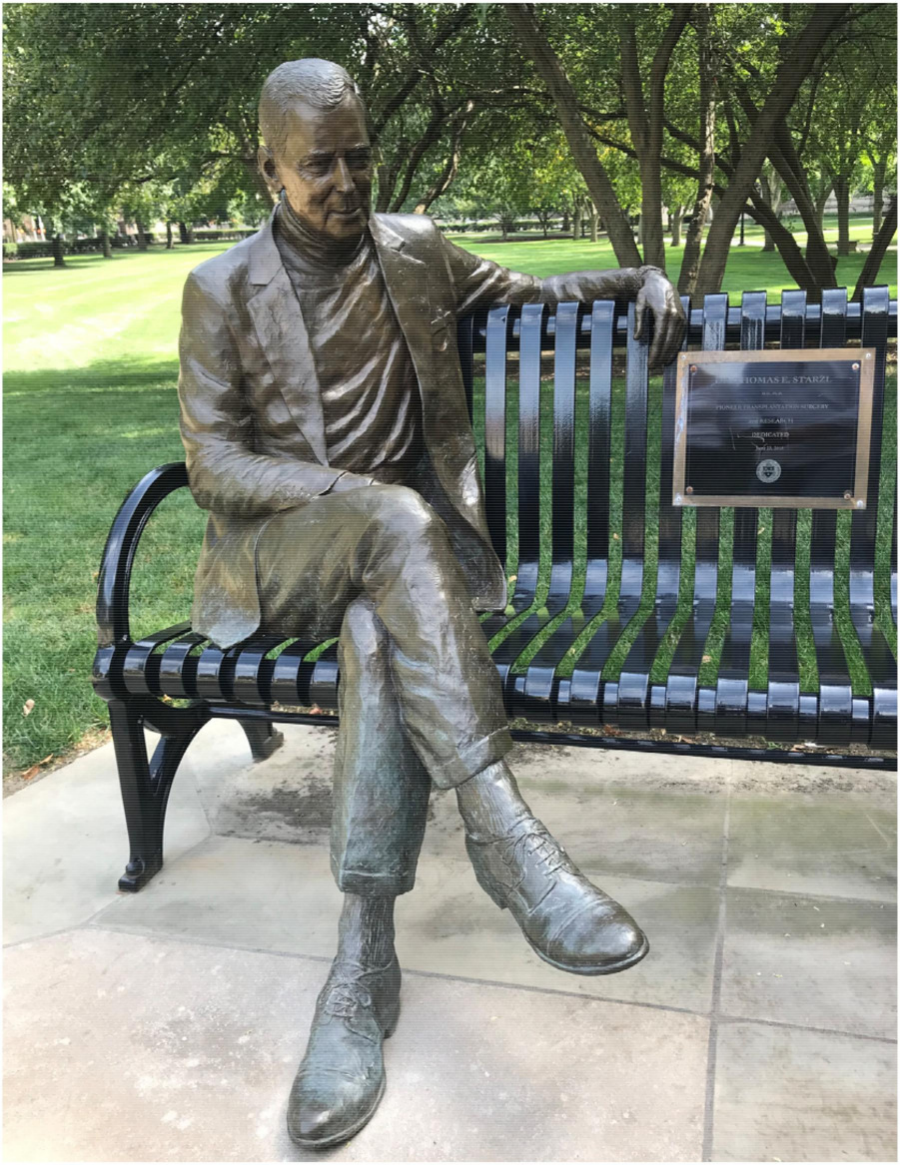
A commissioned statue of Thomas Starzl outside of Heinz Chapel at the University of Pittsburgh.

Dr. Starzl's legacy lives on—in the advances that have aided millions of patients, training thousands of healthcare providers and establishing a discipline that transformed medicine and science. No one had accomplished more, contributed more, sacrificed more to his profession—no one was more dedicated than Thomas Starzl. No one other than Dr. Starzl could have envisioned the path to achieving success in a field that seemed doomed to irrelevance. He was visionary, a brilliant surgeon, scientist, strategist, philosopher and a mentor.

## Data Availability

The original contributions presented in the study are included in the article/Supplementary Material, further inquiries can be directed to the corresponding author.
